# Nanomedicine: A Useful Tool against Glioma Stem Cells

**DOI:** 10.3390/cancers13010009

**Published:** 2020-12-22

**Authors:** Elia Bozzato, Chiara Bastiancich, Véronique Préat

**Affiliations:** 1 Advanced Drug Delivery and Biomaterials, Louvain Drug Research Institute, Université Catholique de Louvain, 1200 Brussels, Belgium; elia.bozzato@uclouvain.be; 2 Institute Neurophysiopathol, INP, CNRS, Aix-Marseille University, 13005 Marseille, France; chiara.bastiancich@univ-amu.fr

**Keywords:** glioblastoma, brain tumor, nanomedicine, cancer stem cell, targeted therapy

## Abstract

**Simple Summary:**

Glioblastoma is one of the deadliest brain cancers, and despite the efforts made in the last few years, the life expectancy of patients is still low. In most cases, even with the best treatments available, the tumor will eventually return. One of the main causes of this appears to be a fraction of cancer cells that are known as glioma stem cells. They have different characteristics than normal cancer cells, and some drugs can eliminate them. However, using such drugs is not always safe or effective, and nanomedicine can have improved effects as well as additional benefits. This review focuses on the nanomedicine strategies that have been employed in the last 5 years and their relative advantages, which make nanomedicine a promising approach for the eradication of glioma stem cells.

**Abstract:**

The standard of care therapy of glioblastoma (GBM) includes invasive surgical resection, followed by radiotherapy and concomitant chemotherapy. However, this therapy has limited success, and the prognosis for GBM patients is very poor. Although many factors may contribute to the failure of current treatments, one of the main causes of GBM recurrences are glioma stem cells (GSCs). This review focuses on nanomedicine strategies that have been developed to eliminate GSCs and the benefits that they have brought to the fight against cancer. The first section describes the characteristics of GSCs and the chemotherapeutic strategies that have been used to selectively kill them. The second section outlines the nano-based delivery systems that have been developed to act against GSCs by dividing them into nontargeted and targeted nanocarriers. We also highlight the advantages of nanomedicine compared to conventional chemotherapy and examine the different targeting strategies that have been employed. The results achieved thus far are encouraging for the pursuit of effective strategies for the eradication of GSCs.

## 1. Introduction

Glioblastoma (GBM) is a grade IV astrocytoma, and the prognosis for GBM patients is very poor. Currently, the standard of care therapy includes surgical resection of the main tumor mass, followed by radiotherapy and concomitant chemotherapy with oral temozolomide (TMZ) [1]. However, this therapy has limited success due to the intrinsic characteristics of the tumor, such as the tumor heterogenicity, development of chemoresistance, and presence of glioma stem cells (GSCs). These factors lead to tumor recurrences. Recently, the overall survival of GBM patients has slightly increased from 16.0 months to 20.9 months with the additional application of tumor-treating fields to the standard of care therapy [2]. Nevertheless, despite this significant improvement, GBM still remains an unmet medical need, and successful long-term therapies urgently need to be found.

GBM is characterized by resistance to treatment and high intertumor and intratumor phenotypic and genetic heterogeneity [3]. Many advances have been made in the past decade to uncover the genetic diversity of GBM and the clone-specific functional profile, showing that even within the same tumor, the combination of various molecular subclasses could be found (e.g., [4,5,6]). This diversity also indicates the presence of GSCs, which are defined as a quiescent subpopulation of cancer cells with high self-renewing abilities that are able to recreate a tumor after transplantation [7]. Even though the precise cell of origin of GBM is still a controversial issue, as some experts contend that it arises from a subpopulation of neural stem cells, while others argue that it arises from the transformation of more differentiated astrocytes [8], it is now recognized that presence of GSCs and crosstalk with their supportive niche contributes to tumor malignancy [9]. Moreover, they are responsible for the onset of tumor recurrence, and therefore, are a promising therapeutic target to prevent GBM relapse. Several publications have recently highlighted how GSC location at the invasive margins, heterogeneity, and dynamism (transcriptional, epigenetic, and metabolic) can play an important role in the response to surgery, radiotherapy, and chemotherapy (e.g., [10,11]). A review from Liu et al. [12] evaluates the potential involvement of brain tumor stem cells in postoperative stem cell niches and their role in tumor relapse, and their input should be considered for the development of adapted nanomedicines. Indeed, while it is true that most nanomedicines are intended for a post-surgical application, most studies report their efficacy on preclinical models designed to treat established GBM. This overlooks the fact that surgical resection of brain tumors can create an environment that can stimulate the proliferation of residual tumor cells (GSCs, tumor microtubes, and infiltrating GBM cells), leading to tumor recurrences. Here, we would like to highlight how nanomedicines can be used to overcome some of the limitations of conventional chemotherapies targeting GSCs, thus representing a promising approach for GBM therapy.

## 2. Glioma Stem Cells

Due to their dormant state, GSCs are intrinsically resistant to conventional chemotherapeutics that act on rapidly proliferating cancer cells, such as alkylating agents, antimetabolites, and mitotic inhibitors. Furthermore, they can actively resist chemo- or radiotherapy by the activation of checkpoint mechanisms, in order to recover efficiently from the genotoxicity induced by the therapy. Another mechanism of resistance for GSCs is the expression of drug efflux mechanisms (ABC transporters) to protect the cells from xenogeneic molecules [13]. Autophagy, which is required for stemness maintenance, not only in normal tissue stem cells but also in GSCs, has been shown to contribute to therapy resistance [14]. Moreover, the Notch signaling pathway is involved in the resistance of GSCs to radiotherapy. The inhibition of this pathway through γ-secretase inhibitors is able to induce radiosensitivity by targeting the subpopulation of cells that bears the GSC marker CD133 [15].

GSCs are also characterized by specific pathways that are implied in the conservation of stemness characteristics or in tumor formation. The Notch pathway can inhibit cell differentiation and therefore maintain the stem-like properties of GSCs [16]. In patient-derived GSCs taken from the periphery of the tumor, Hu and collaborators demonstrated that Notch promotes self-renewal and inhibits differentiation [17]. In recurrent GBM samples, CD133, Notch, and VEGF expression was higher after radiotherapy and chemotherapy, and after a second surgery and treatment with bevacizumab, the overall survival was significantly longer for Notch-negative patients [18]. Furthermore, cells from the interface region are CD133^+^/Notch1^+,^ and there is a positive-feedback loop between NOTCH1 and SOX2 [19]. The aberrant activation of Wnt signaling causes the transcription of c-Myc and other target genes leading to tumor formation [20]. It also participates in the maintenance of stemness characteristics by regulating the expression of PLAGL2 (pleiomorphic adenoma gene-like 2) that is able to suppress the differentiation of GSCs [21]. Finally, the Sonic Hedgehog (Shh) pathway is essential for cell survival and sustained growth of the tumor. In fact, it regulates the expression of stemness genes in glioma GSCs [22].

**Figure 1:**
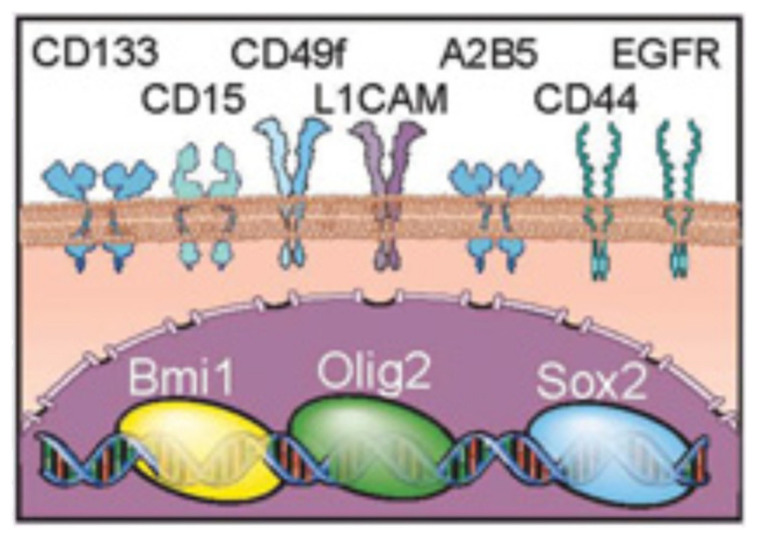
Intracellular and extracellular glioma stem cell (GSC) markers. Adapted from [26].

GSCs can be isolated from cancer cells and tissue stem cells using specific intracellular or extracellular markers (Figure 1), although functional validation should also be employed to assess the stem cell characteristics (self-renewal and tumor formation) [8]. The most common marker is CD133 or Prominin-1, a transmembrane glycoprotein that is also expressed by human neural stem cells [23]. However, evidence also suggests the existence of CD133^-^ GSCs [24], and therefore, a single marker cannot automatically identify GSCs. Other common markers are A2B5, a glycolipid found on the cell surface of oligodendrocyte progenitors; stage-specific embryonic antigen-1 (SSEA-1, also known as CD15) an embryonic antigen with a carbohydrate structure; and Nestin, a filament protein that is also expressed by neural progenitor cells [25]. Additionally, high ALDH-1 (aldehyde dehydrogenase 1) activity and the high extrusion of xenobiotics through ABC transporters are two functional markers that have been associated with GSCs [25].

The metabolism of GSCs is very plastic. In fact, the dependence on oxidative or nonoxidative metabolism is heterogeneous throughout the tumor. Fast-dividing cells rely more on anaerobic glycolysis [27], creating the Warburg effect as an adaptation metabolism for their rapid growth. In an acidic environment, GSCs can undergo mesenchymal differentiation, resulting in an increase of therapy resistance [28]. On the other hand, slowly proliferating cells are more dependent on oxidative phosphorylation (OXPHOS) and lipid oxidation, and GSCs in particular can metabolize various substrates, making it difficult to find a pharmacological target [10]. GSCs have been reported to have lower glucose consumption than normal GBM cells [10]. However, depending on their microenvironment, they are able to adapt to nutrient and stress conditions by increasing their glycolytic activity [10]. In fact, GSCs can also upregulate high-affinity transporters, such as GLUT3, to obtain sufficient nutrients and support their rapid metabolism [10].

GSCs can adapt and are able to interact with different niches. For example, GSCs that are located at the perivascular niche are in contact with the endothelium that secretes ligands that bind to the transmembrane Notch receptor on GSCs, leading to the activation of the Notch pathway and supporting GSC self-renewal. In exchange, GSCs can transdifferentiate into pericytes to contribute to the vascular structure, thus promoting tumor growth [26]. GSCs can also interact with immune cells through their metabolism. They can regulate the microenvironment and generate stress for immune cells, thus creating a globally suppressive tumor microenvironment that allows for immune escape and tumor progression [10]. In return, macrophages, which are the most represented type of tumor-infiltrating cell, participate to the regulation of GSC metabolism by increasing their fatty acids synthesis and trafficking, thus promoting lipid oxidation, which is one of the main metabolic pathway of GSCs [10]. Moreover, through the secretion of interleukin 10 (IL-10) and transforming growth factor beta (TGF-β), GSCs are able to suppress the tumor-associated microglia, generating an M2 immunosuppressive phenotype [26]. Furthermore, GSCs are able to regulate immune cells directly, causing the activation of regulatory T cells, the inhibition of cytotoxic T cell proliferation, and the induction of cytotoxic T cell apoptosis [29,30].

GSCs however are not a static, discreet cell subpopulation; their stemness is rather a dynamic and reversible state. There is considerable evidence that EMT (epithelial to mesenchymal transition) is involved in the dynamism of GSCs [31], and that various factors can stimulate or revert this transition [32,33,34]. Furthermore, based on their location in the tumor, they can have different characteristics and exert different functions: while GSCs in the core hypoxic regions support proliferation and therapy resistance, GSCs from the outer invasive region are enriched for their invasive potential and promote tumor recurrence after resection [11].

## 3. Chemotherapy against GSCs

**Figure 2:**
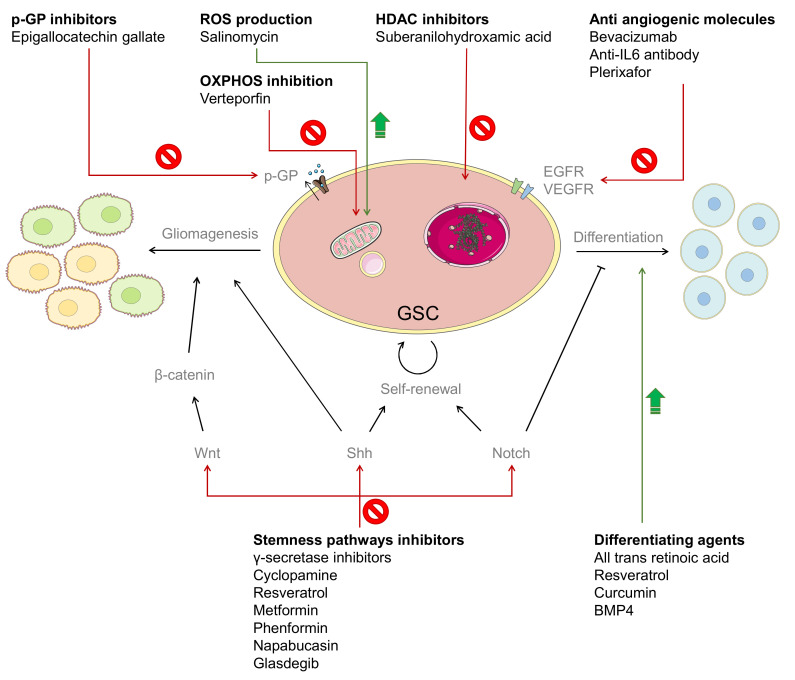
Anti-GSC molecules and their mechanisms of action.

Despite the high number of researchers and clinicians investigating GBM, treatment options for this tumor have remained nearly unchanged for the last 15 years [35]. Some progress has been made in the field of personalized therapy, thanks to the ChemoID assay, which consists of a viability test on GSCs and bulk tumor cells from freshly resected samples, in order to identify the most effective drug or combination of drugs. Patients were therefore treated with the selected drugs, and 12 out of 14 cases had complete or at least partial response to the therapy [36]. In order to better relate to intra-tumor heterogeneity, this same approach could be used on samples obtained from different tumor regions from each patient. After the viability assay on GSCs from each sample, the patient could be treated with the combination of drugs that demonstrated cytotoxicity in the different regions. However, the study from Ranjan et al. [36] suggests that, along with chemotherapy directed against GBM cells, combination therapies also targeting GSCs could be necessary. The possible approaches that can be adopted in order to eliminate GSCs are represented in Figure 2.

One of the strategies that has been explored to attack the GSC population is to inhibit specific GSC pathways, such as Notch, Wnt, and Shh. For example, the inhibition of Notch activation through γ-secretase inhibitors is reported to reduce the CD133-positive GBM cell population in vitro and to reduce tumorigenicity of pretreated brain tumor cells subcutaneously injected in athymic mice [37]. Cyclopamine, a Shh inhibitor, was able to reduce neurosphere formation and block the tumor formation of intracranially injected GSC cells [38]. Resveratrol can modulate the Wnt pathway and decrease the proliferation and mobility of GSCs [39]. Metformin can inhibit AKT signaling, which is involved in the response to stress conditions to promote GSC growth and survival [40]. Its analog Phenformin is also able to inhibit the self-renewal of GSCs, thus reducing the growth of xenograft tumors and prolonging mice survival [41]. Napabucasin, a STAT3 inhibitor, can inhibit the expression of stemness-associated genes and the growth of GBM spheroids in vitro [42], and has led to the loss of GSCs associated genes, induction of apoptosis, and inhibition of in vivo tumor growth of GSCs derived from recurrent GBM [43]. This drug has also been used in a phase I/II clinical trial in combination with TMZ [44]. Glasdegib and RO4929097, a Shh pathway inhibitor and a γ-secretase inhibitor, respectively, are also being used in combination with TMZ in two different ongoing clinical studies [45,46].

GSCs are also implied in therapy resistance, and they can actively participate to this process though mechanisms like DNA repair, pro-surviving signaling, and most importantly, drug efflux [47]. Therefore, another approach is to employ P-gp (permeability glycoprotein) or to induce the differentiation in normal GBM cells, in order to sensitize them to conventional chemotherapy. It has been demonstrated that CD133 contributes to the regulation of MDR1 through the phosphoinositide 3-kinase (PI3K)- or Akt–NF-κB signal pathway [48]. Moreover, the invasive margin of GBM displays an increased expression of ABCG2 [49], which is another efflux pump belonging to the ABC transporters superfamily. It has been shown that reduction in ABCG2 expression can decrease the cell migration and invasion of GSCs [50]. An example of P-gp inhibitor is epigallocatechin gallate, which was able to reduce the P-gp expression and neurosphere formation of GSCs obtained from the U87 cell line, and increase the sensitivity of these cells to TMZ [51]. The differentiating agent transretinoic acid was able to deplete GSC markers and reduce the formation of neurospheres, and the effect on cell migration was improved in combination with rapamycin [52]. Resveratrol can induce the degradation of Nanog, which is essential for stemness maintenance, thus leading to the loss of GSC markers and decreased tumorigenicity [53]. Curcumin was demonstrated to activate autophagy, thus triggering the differentiation cascade of GSCs and causing a decrease in its self-renewal and clonogenic abilities [54]. Finally, bone morphogenetic protein 4 (BMP4) is commonly used to reduce the number of GSCs by inducing their differentiation, and therefore increasing the response to conventional therapies [55]. BMP4 is also currently being administered through convection-enhanced delivery (CED) in a phase I clinical trial [56].

Additionally, tackling the tumor microenvironment through antiangiogenic or antivasculogenic molecules can also decrease the number of GSCs. The treatment with bevacizumab was able to reduce the number of CD133^+^/Nestin^+^ cells, along with reducing the microvasculature density and tumor growth in U87 glioma xenografts [57]. Moreover, the administration of antibodies against a proangiogenic factor like IL-6 could delay the growth of tumors obtained by the injection of GSCs in a xenograft model [58]. Another antivasculogenic molecule, the biciclame compound plerixafor (AMD3100), was able to inhibit irradiation-induced vasculogenesis in vivo by preventing the binding of the chemokine stromal cell-derived factor 1 (SDF-1, involved in the migratory process of GBM) to its receptor C-X-C chemokine receptor type 4 (CXCR4) [59].

Targeting the DNA methylation of GSCs through histone deacetylase inhibitors (HDAC) inhibitors is another strategy that has been described in the literature. In fact, suberanilohydroxamic acid (SAHA) is able to induce autophagy in GSCs, thus leading to decreased cell viability in vitro and reduced tumor growth in vivo [60]. 

Finally, salinomycin has been used on GBM cells in combination with HDAC inhibitors, such as valproate and vorinostat [61], and it has also shown anti-CSC activity in other cancer types [62]. Even though its mechanism of action needs to be elucidated, it has been reported that it can induce ROS production in GSCs, thus leading to endoplasmic reticulum stress and cell death via regulated necrosis [63]. Additionally, verteporfin can target the mitochondria of GSCs and inhibit OXPHOS without any toxicity to normal cells [64].

In many cases, the elimination or impairment of GSCs has led to decreased tumor growth and increased survival in preclinical in vivo models, highlighting once again the importance of tackling GSCs in the treatment of GBM. However, only a few of the abovementioned molecules are being tested in clinical trials (mostly GSC pathway inhibitors), and the results are not yet available.

## 4. Nanomedicine against GSCs 

### 4.1. Nanomedicine for GBM Treatment

The intrinsic limits of chemotherapy are the lack of specificity, harmful side effects, low therapeutic index, and transport limitations [65]. Indeed, many drugs, including those cited in the previous chapter, have poor solubility, high toxicity due to the uncontrolled drug biodistribution, or poor stability in the physiological environment. Moreover, when administered systemically, they need to cross the blood–brain barrier (BBB) to reach the GBM tumor site at therapeutic concentrations, often leading to severe, dose-related systemic side effects. Some drugs are not stable in biological fluids and have a very short half-life; therefore, multiple administrations are required to achieve the therapeutic concentration at the tumor site, reducing patient compliance. 

Nanomedicine can help provide a solution for these problems. The encapsulation of drugs in nanosized carriers can protect them from degradation, increase the amount of drug reaching the tumor site, and decrease the intensity of the side effects, thus increasing the safety of the treatment. The maintenance of a correct therapeutic level can be facilitated by the controlled release of the drug over time. Moreover, the surface of the nanocarrier can be suitably modified with targeting moieties in order to actively and specifically recognize GBM cells and GSCs, or to cross the BBB more easily. This can further increase the uptake of the nanoparticles (NPs) by GSCs and enhance their residence time in the tumor.

The BBB is a natural barrier that protects the central nervous system from exogenous compounds or macromolecules. Even though in GBM the patients’ BBB parts are disrupted and leaky [66,67], the crossing of the BBB still represents a challenge for GBM treatment, due to the poor blood perfusion and the high interstitial pressure. The BBB can be bypassed by administering drugs locally, through implants or CED. A local delivery has the advantage of increasing the drug concentration in its site of action while minimizing the side effects. However, systemic delivery is still the preferred strategy for inoperable tumors, and thanks to its being less invasive, also allows for the administration of multiple doses.

Herein, we review the nanomedicine approaches that have been developed in the last 5 years against GSCs, dividing them by nontargeted and targeted systems (Table 1 and Table 2, respectively). 

### 4.2. Non-Targeted Nanomedicines

**Figure 3:**
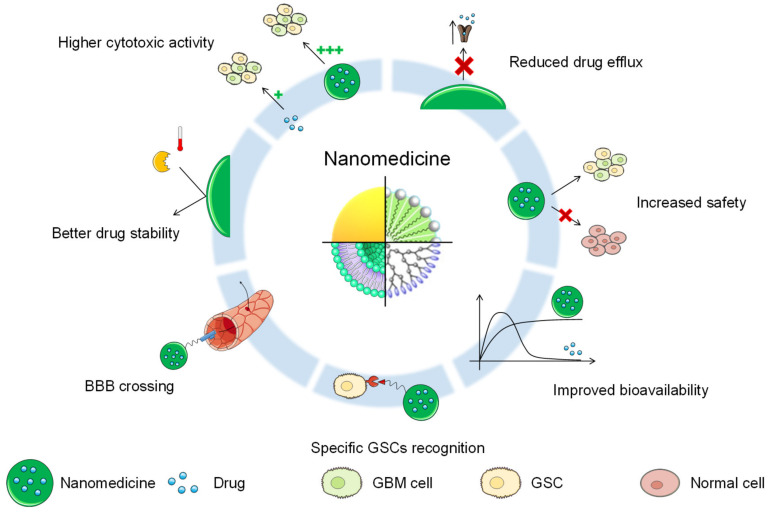
Potential advantages of nanomedicine against GSCs.

NPs can exploit the enhanced permeation and retention (EPR) effect to accumulate and increase their residency time at the tumor site [92,93]. The EPR effect consists of the preferential accumulation of NPs in the tumor site caused by two components: (i) due to their rapid growth, blood vessels in the tumor present a leaky and less organized structure than normal blood vessels; and (ii) inefficient lymphatic drainage. However, in the past few years, due to its intratumor and intertumor variability, together with the differences between animal models and patients, the EPR effect has been questioned [94,95]. Despite this controversial topic, in order to eliminate GSCs, nanomedicine can still offer many advantages when compared to conventional chemotherapy (Table 1, Figure 3).

One of the advantages of using a drug delivery system is the increase in safety compared to the free drug. For example, paclitaxel-loaded chitosan NPs covered with 1,3β-glucan were demonstrated to have a lower half maximal inhibitory concentration (IC50) value than the free drug on C6-derived stem-like cells, and significantly lower hemolytic activity than the drug suspension [96], thus showing an increased safety profile. Cytarabine-loaded liposomes showed an increased safety profile compared to the free drug [97]. This formulation is currently being examined in a phase I/II clinical trial [98], and is reported to tackle the subventricular zone, which is one of the proposed sites of origin for GSCs [99]. 

Another advantage of nanomedicine is the increased stability. The encapsulated molecule can be protected from degradation processes, such as hydrolysis, enzymatic degradation, or metabolism. This is usually the case for nucleic acids, such as miRNAs and siRNAs, as their blood half-life is very low. Various types of nucleic acids have been encapsulated in polymeric NPs [69,74], lipid–polymer NPs [73], superparamagnetic iron oxide NPs [71,76], and gold NPs [72,100]. These formulations were able to increase the internalization of the nucleic acid by passive targeting, inducing an efficient silencing of GSC-related genes, reducing GSC proliferation and invasion, and prolonging animal survival in vivo.

Moreover, encapsulation in a drug delivery system can also reduce the efflux of the drug. Unlike free drugs, which enter the cells through diffusion and locate near the efflux pumps, nanomedicines enter the cells through endocytosis and are transported into the cell via endo-lysosomal trafficking, preventing them from being a substrate for drug efflux pumps [101]. Etoposide, which is an efflux pump substrate, was loaded in layered double-hydroxide nanocomposites, thus prolonging its retention time in the cells and increasing its accumulation in the tumor site. This brought about the elimination of GSCs in vitro and decreased tumor growth in the xenograft mouse model [68].

Nanomedicine can also improve the bioavailability of molecules like curcumin. Curcumin was formulated in liposomes in combination with epicatechin gallate and resveratrol, and after intraperitoneal injection, it obtained an almost constant plasma concentration, which led to increased mouse survival in the in vivo experiment. Furthermore, this liposomal formulation was able to decrease the GSC subpopulation of GL261 cells [70].

Additionally, even though this advantage is less common than others, drug delivery systems can in some cases increase the activity of the drugs. Atorvastatine-loaded polymeric micelles were indeed able to inhibit the growth of CSC spheroids compared to the single drug [102]. In the case of zinc-doped copper oxide nanocomposites, the NPs have an intrinsic inhibitory effect, decreasing the colony formation of TMZ-resistant GSCs, but at the same time exerting lower toxicity on normal cells [75].

### 4.3. Targeted Nanomedicines

The design of nanosystems can be implemented by the addition of a targeting agent, usually an antibody or a ligand, that selectively recognizes cell surface markers overexpressed in a certain population. This has the aim of making the carrier interact with the cell surface, and thanks to the interaction, induce its cellular uptake by endocytosis, ultimately acting as a Trojan horse and releasing its cargo directly inside the cell. Therefore, targeted nanomedicines have the advantage of increasing the amount of cytotoxic agent inside the target cell, reducing the proportion of drug that is delivered to healthy tissues.

**Figure 4:**
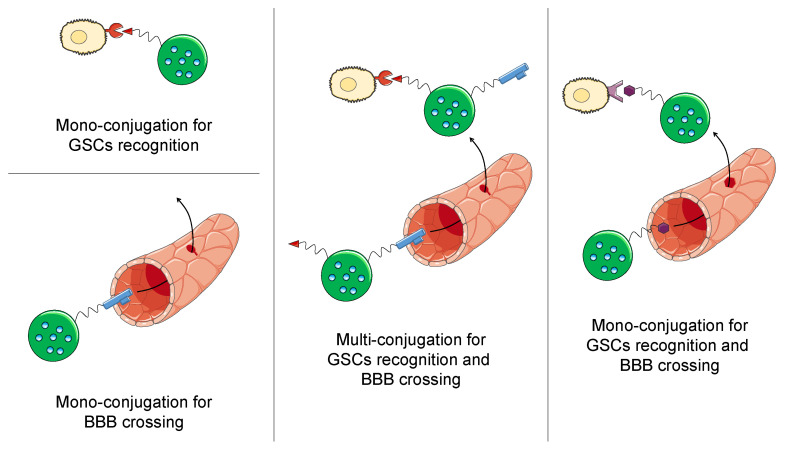
Targeting strategies employed to reach GSCs.

Different strategies have been employed to specifically target GSCs (Table 2, Figure 4), and the most common and straightforward is the use of antibodies against CD133, which is the most described GSC marker in the literature. The conjugation of anti-CD133 antibodies to polymeric dendrimers loaded with mercaptoundecahydrododecaborate, a substance employed in boron neutron capture therapy, has led to significantly increased drug uptake and the decreased clonogenic survival of CD133+ cells after neutron radiation. This also produced significantly prolonged mouse survival in an orthotopic xenograft model [88]. Anti-CD133 antibodies were also used as carriers and targeting agents at the same time. IR700, an agent employed in near-infrared photoimmunotherapy, was conjugated to the antibody with a theranostic application. The authors successfully detected CD133+ cells following intravenous administration and laser irradiation in mice bearing orthotopic brain tumors initiated from patient-derived GSCs, and at the same time observed extended overall survival [84].

Another common strategy that has been adopted is the conjugation of anti-transferrin receptor (anti-TfR) antibodies. Resveratrol-loaded targeted liposomes are capable of reducing the growth of glioma neurospheres. Moreover, the targeted formulation has shown a significantly increased association with glioma neurospheres compared to the nontargeted liposomes [103]. In addition, targeted polymeric NPs were conjugated to antisense oligonucleotides against laminin-411, which is correlated to GSC marker expression. This nanosystem was able to reduce the protein expression and prolong the survival of mice intracranially transplanted with LN229 and U87 MG cells [78].

Another approach that has been applied is the use of the anti-EGFR antibody. Cetuximab was bound to iron NPs, and showed enhanced uptake by EGFR- and EGFRvIII-expressing GSCs and neurospheres, as well as a significantly increased animal survival in vivo [87].

One of the main obstacles that nanomedicine encounters in the treatment of GBM is the crossing of the BBB, whose natural function is to prevent exogenous structures from reaching the brain. Consequently, nanocarriers for GBM must be designed to cross the BBB and reach the tumor site in higher amounts. The cyclic RDG peptide was linked to micelles loaded with an antisense nucleotide against TUG1, a gene participating in Notch signaling. The formulation in a targeted micellar delivery system allowed the crossing of the BBB and the accumulation in the tumor site, thus enhancing TUG1 silencing in a mouse xenograft model [91].

Several authors developed multifunctional nanocarriers by combining the targeting of GSCs and the crossing of the BBB. TMZ-loaded liposomes were conjugated with an anti-CD133 antibody for targeting GSCs and angiopep-2 for BBB crossing. Angiopep-2 can bind to the low-density lipoprotein (LDL) receptor-related protein, which is highly expressed on the endothelium of the BBB. This system was able to bind to GSCs more efficiently than the nontargeted system, and showed an increased permeability of the BBB in vitro. Moreover, the dual-targeted liposomes were able to decrease the tumor size and prolong the mice survival in the orthotopic, in vivo GSC model [90]. Paclitaxel and surviving siRNA-loaded liposomes were also conjugated with an anti-CD133 aptamer for targeting GSCs and Angiopep-2 for crossing the BBB. Targeted liposomes had an improved uptake in cancer stem cells compared to the nontargeted ones. Moreover, while Taxol and nontargeted liposomes had almost the same effect, targeted liposomes produced a significant decrease in the cell viability of CD133+ cells. The formulation was also able to significantly reduce tumor growth and prolong mouse survival in vivo [83].

Finally, the same targeting moiety can be employed for both targeting GSCs and crossing the BBB. A mannose derivative, p-aminophenyl-α-d-mannopyranoside, was used to functionalize curcumin- and quinacrine-loaded liposomes. Compared to the nontargeted one, this nanocarrier was able to cross a BBB in vitro model more efficiently and significantly increase the uptake in GSCs. Moreover, the targeted liposomes could increase the median survival and inhibit the tumor growth of tumor-bearing mice [89]. Surprisingly, the anti-TfR antibody has also been demonstrated to exert both functions in p53-loaded liposomes. This formulation was also capable of crossing the BBB and targeting GSCs in vivo. Moreover, the delivery of a p53-encoding plasmid was able to decrease the expression of O^6^-methylguanine-DNA methyltransferase (MGMT), thus increasing the sensitivity of the cells to TMZ. Due to the promising preclinical results, this formulation is currently under investigation in a phase II clinical study; however, no results have been released yet [80,81,104].

## 5. Conclusions

Despite extensive research, the need for an efficient, long-term treatment against GBM remains high. As GSCs play a major role in GBM recurrence and resistance to treatment, it is important to take them into account and include anti-GSC molecules in combination regimens to increase their therapeutic benefit. In this review, we have examined the nanosystems that have been developed and used against GSCs in the past 5 years (Table 1 and Table 2), trying to highlight their advantages compared to conventional chemotherapeutic treatments. Surprisingly, most of the delivery systems reported in the literature have been developed for systemic administration, while the use of local delivery systems, which have the advantage of bypassing the BBB and delivering high drug concentrations at the tumor site, are poorly represented. In our opinion, a suitable delivery system should be adaptable to the resection cavity to ensure adhesion to the brain tissue, thus delivering the drug(s) in the regions where recurrence is more probable. In fact, most of the recurrences arise nearby the resection cavity [105]. Moreover, this delivery system should include multiple drugs, at least one directed against normal GBM cells and at least one directed against GSCs, as the combination therapy approach is considered promising and is being tested in various clinical trials [106]. Finally, the drug(s) should preferentially be released from the delivery system in a sustained way, in order to maintain a therapeutic drug concentration at least until the beginning of the conventional radio- and chemotherapy (or even beyond, provided that none of the drugs interact with TMZ in an antagonistic manner). However, only a few of the nanomedicine systems included in this review have reached the clinical stage up to now, and therefore, there is still considerable research to be performed in order to explore new potential routes or consolidate established nanomedicine strategies. However, nanomedicine can be a promising strategy for adjuvant GBM therapies, in order to eliminate the GSC population and eradicate these deadly tumors.

## Figures and Tables

**Table 1:** Nontargeted nanosystems for the treatment of preclinical glioblastoma (GBM).

Molecule(s)	Nanoparticle	Cell Line(s)	Preclinical Model	Outcome	References
Etoposide	Layered double hydroxide nanocomposites	U87 MG U87 MG-derived GSCs	Nude mice, hypodermically injected GSCs, treated by i.p. injection	GSC elimination Downregulation of pluripotency genes Decreased tumor growth Increased drug accumulation	[68]
miR-148a miR-296-5p	Cationic polymeric NPs	GBM1A	Orthotopic human GBM xenografts, treated by intracranial infusion	Lower expression of GSC-correlated genes ~70% animal survival	[69]
Curcumin Epicatechin gallate Resveratrol	Liposomes	GL261	Orthotopic syngenic mice, treated by i.p. injection	Decrease of CD133+ and SOX2+ cells Constant plasma concentration Increased mice survival	[70]
HOTAIR-siRNA	SPIONs	SHG44	Subcutaneous injection of pretreated human GSCs in nude mice	Inhibition of CD133+ cell proliferation	[71]
miR-182	Gold NPs	Patient-derived cells U87 MG	Orthotopic xenograft model, treated by i.v. injection	Higher animal survival	[72]
siRNA	Lipopolymeric NPs	Patient-derived cells	Orthotopic xenografts, treated by intracranial injection or intracranial infusion	Knock-down of CSC-related markers Extension of the median survival	[73]
GLUT3 siRNA	PEG–PLA NPs	U87 MG U251	Subcutaneous human glioma xenograft, treated by i.v. injection	Increased the internalization Reduction of tumor growth and CSC markers	[74]
Zinc-doped copper oxide nanocomposites TMZ *	Zinc-doped copper oxide nanocomposites	C6 U87 U251 A172	Subcutaneous GBM xenografts, treated by i.t. injection	Higher cytotoxic effect Reduction of sphere and colony formation	[75]
microRNA-374a overexpression plasmid	SPIONs	Patient-derived CD133+ GBM cells	Subcutaneous injection of pretreated human GSCs in nude mice	Decreased proliferation rate and invasiveness of CD133+ cells Tumorigenicity inhibition	[76]
Iguratimod	PLGA NPs	U87 U118 U251	Subcutaneous xenograft model, treatment by i.v. injection	Cell growth inhibition Sphere formation inhibition Decreased tumor growth	[77]

Legend: * free drug. Abbreviations: HOTAIR: HOX transcript antisense RNA; TMZ: Temozolomide; NPs: nanoparticles; SPION: superparamagnetic iron oxide NPs; GSCs: glioma stem cells; CSCs: cancer stem cells; i.p.: intraperitoneal; i.v.: intravenous.

**Table 2:** Targeted nanosystems for the treatment of preclinical glioblastoma.

Molecule(s)	Nanoparticle	Targeting	Cell Line(s)	Preclinical Model	Outcome	References
Antisense oligonucleotides targeting laminin-411	Polymeric nanoconjugate	anti-TfR receptor antibodies	U87 MG LN229 Patient-derived cells	Orthotopic xenograft model, treatment by i.v. injection	Reduced protein expression Prolonged mouse survival	[78]
Antisense oligonucleotides targeting CK2α and EGFR/EGFRvIII	Polymeric nanoconjugate	anti-TfR mAb anti-EGFR mAb cetuximab	U87 MG LN229	Orthotopic xenograft model, treatment by i.v. injection	Lower CSC marker expression Improved survival	[79]
p53encoding plasmid TMZ *	Cationic liposomes	anti-TfR antibody	U87 T98G LN-18 U87–luc2 U251	Subcutaneous and orthotopic xenograft models, treatment by i.v. injection	Cell sensitization to TMZ Tumor growth reduction Mean survival increase	[80,81]
Bevacizumab Chloroquine	Bevacizumab	Bevacizumab	U87 Primary GBM specimens	Orthotopic injection of GSCs, treatment by i.p. injection	Decreased tumor growth Improved overall survival	[82]
Paclitaxel Survivin siRNA	Cationic liposomes	Angiopep-2 A15	U251–CD133- U251–CD133+	Orthotopic xenograft model, treatment by i.v. injection	Improved uptake of CSCs Decreased CD133+ cell viability Tumor growth reduction Prolonged mouse survival	[83]
IR700	Anti-CD133 antibody	Anti-CD133 antibody	CD133–OE U251 NCH421k GBM-SC	Subcutaneous and orthotopic xenograft models, treatment by i.v. injection	Extended overall survival	[84]
Paclitaxel	Liposomes	Octa-arginine-conjugated cyclic RGD	C6	Orthotopic injection of C6 cells, treatment by i.v. injection	Induction of apoptosis on C6 stem cells Improved mice survival Better safety profile	[85]
Vinorelbine Tetrandrine	Liposomes	Polyethylenimine Vapreotide	C6 GSCs	Orthotopic injection of GSCs, treatment by i.v. injection	Higher cytotoxic effect Higher antitumor efficacy	[86]
Cetuximab	Iron oxide NPs	Cetuximab (anti-EGFR antibody)	U87 MG U87 MGwtEGFR LN229wtEGFR Patient-derived cells	Orthotopic xenograft model, treatment by CED infusion	Enhanced cytotoxicity Improved animal survival	[87]
Mercaptoundecahydrododecaborate	polyamido amine dendrimers	Anti-CD133 antibody	SU2 U87	Orthotopic xenograft model, treatment by i.t and/or i.v. injection	Increased uptake Decreased clonogenic survival Prolonged survival	[88]
Curcumin Quinacrine	Liposomes	p-aminophenyl-α-d-mannopyranoside	C6	Orthotopic injection of GSCs, treatment by i.v. injection	Higher growth inhibition for CSCs Higher efficacy of the combination	[89]
TMZ	Liposomes	Angiopep-2 Anti-CD133 antibody	U87 MG	Orthotopic xenograft model, treated by i.v. injection	Increased cytotoxicity Decreased tumor size Prolonged mice survival	[90]
Antisense oligonucleotides	Polymeric micelles	Cyclic RGD	Patient-derived GSCs	Orthotopic xenograft model, treated by i.v. injection	Induction of apoptosis Accumulation in the tumor site Enhanced TUG1 silencing	[91]

Legend: * free drug. Abbreviations: CK2α: protein kinase CK2 catalytic α subunit; EGFR: epidermal growth factor receptor; TMZ: Temozolomide; NPs: nanoparticles; TfR: transferrin receptor; RGD: Arginyl-glycyl-aspartic acid peptide; GSCs: glioma stem cells; CSCs: cancer stem cells; i.p.: intraperitoneal; i.v.: intravenous.
